# Cambrian explosion fossils from the North China craton

**DOI:** 10.1093/nsr/nwac082

**Published:** 2022-04-29

**Authors:** Jean-Bernard Caron

**Affiliations:** Department of Natural History, Royal Ontario Museum, Canada

Our understanding of the Cambrian explosion has relied heavily on a few exceptional fossil deposits preserving soft-bodied animals, the so-called Burgess-Shale-type deposits, or BSTs for short. BSTs are named after the famous Canadian fossil deposit discovered by Charles Walcott in 1909. Regardless of where they come from or their age, all BSTs preserve soft-bodied fossils as carbonaceous remains. The distribution of these sites [1] and the number of taxa recovered at each one vary greatly owing to local biostratinomic controls [2], differences in sampling efforts and community variations over temporal and stratigraphic scales [3,4].

Although ∼20 major BSTs are now known globally, most BSTs are still restricted to two major terranes: Laurentia and South China. These terranes represent stratigraphically separate units, of which the most famous and diverse BSTs are the Burgess Shale in Canada and the Chengjiang biota, discovered in South China in 1984. The rarity of BSTs, particularly high-diversity BSTs, as well as of contemporaneous BSTs from other terranes, limits our ability to investigate global temporal and paleobiogeographic patterns of soft-bodied faunal assemblages more thoroughly.

New research by Sun *et al*. formally describes the faunal composition of a new middle Cambrian (Drumian) BST, the Linyi Lagerstätte, named after the town of  Linyi in Shandong Province [5]. This finding is significant because it is from the North China terrane and it is the youngest of all Cambrian BSTs found in China so far, equivalent in age to the Wheeler Formation in Utah (USA). The Linyi Lagerstätte also nicely complements earlier reports of BST fossils such as *Isoxys* [6], *Sidneyia* [7] and *Cambroraster* [8] found near Weifang, a few dozen kilometers to the north of Linyi in Shandong Province. Belonging to the underlying Mantou Formation, this BST is still awaiting a proper quantitative investigation of its faunal assemblage.

The reported taxonomic diversity and quality of preservation of fossils from the Linyi Lagerstätte (N = 35) is certainly unremarkable when compared to the Burgess Shale or the Chengjiang biota, but considering that this study is based on limited samples (ca. 3000 specimens) from just a few small recent excavations, the Linyi Lagerstätte holds great promise for new discoveries.

Among the most remarkable soft-bodied species discovered at the Linyi site are arthropods, including *Cordaticaris striatus*, a newly described radiodont [9]. The Linyi Lagerstätte also includes two species, including one new species, of the very rare arthropod *Thelxiope.* Only a handful of specimens of this genus are known globally. Although the specimens recovered so far do show new features, but are not very well-preserved, there is hope that future specimens will yield more information.

The detailed approach that the authors took in documenting this new BST, counting all specimens from recent systematic excavations, allowed them to compare the Linyi Lagerstätte to other BSTs using quantitative analyses. Based on the preliminary results, the authors hypothesized that the Linyi Lagerstätte represents an intermediate fauna between East Gondwana and Laurentia—not surprising, considering that this fauna lived on a separate terrane between the two. The work on the Linyi Lagerstätte exemplifies the importance of continuing both well-conducted field explorations and detailed investigations to fill in important gaps in our knowledge of the evolution of the first animals.

***Conflict of interest statement***. None declared.
